# Mild reinfection with severe acute respiratory syndrome coronavirus 2 (SARS-CoV-2) Delta variant: First case report from Indonesia

**DOI:** 10.3389/fmed.2022.906469

**Published:** 2022-07-22

**Authors:** Naniek Isnaini, Yan Mardian, Dewi Lokida, Fajar Budiono, Deni P. Butar-butar, Dona Arlinda, Gustiani Salim, Herman Kosasih, Wahyu Nawang Wulan, Jacqueline Perodin, Aaron Neal, H. Clifford Lane, Muhammad Karyana

**Affiliations:** ^1^Tangerang District Hospital, Tangerang, Indonesia; ^2^Indonesia Research Partnership on Infectious Disease, Jakarta, Indonesia; ^3^National Institute of Health Research and Development, Ministry of Health, Jakarta, Indonesia; ^4^Leidos Biomedical Research, Frederick, MD, United States; ^5^National Institute of Allergy and Infectious Diseases, National Institutes of Health, Bethesda, MD, United States

**Keywords:** case report, reinfection, COVID-19, Indonesia, Delta variant

## Abstract

**Background:**

Reinfection with SARS-CoV-2 has been well documented, yet little is known about the degree of protection a previous infection provides against reinfection, especially against Variants of Concern (VOC).

**Case presentation:**

Here we describe a case of an unvaccinated 49-year-old man who experienced two sequential SARS-CoV-2 infections with two different variants, as evidenced by genomic sequencing. The first episode was caused by the Pango lineage B.1.466.2 and resulted in severe COVID-19 with 5 days in an intensive care unit (ICU). The second episode occurred approximately 6 months later, during the Delta surge in Indonesia. Genomic analysis showed that the second infection was caused by the Delta variant (Pango lineage B.1.617.2) and resulted in mild disease that did not require hospitalization. No SARS-CoV-2 nucleic acid was detected between the two episodes, but both binding and neutralizing antibodies to SARS-CoV-2 were detected prior to the reinfection, with the second infection leading to an increase in the levels of antibody.

**Conclusion:**

We confirmed that the patient experienced a reinfection instead of persistent viral shedding from the first infection based on epidemiological, clinical, serological, and genomic analyses. Our case supports the hypothesis that SARS-CoV-2 reinfection may occur once antibody titers decrease or following the emergence of a new variant. The milder presentation in the patient’s second infection deserves further investigation to provide a clear picture of the role of post-infection immunity in altering the course of subsequent disease.

## Introduction

According to WHO data as of June 12, 2022, severe acute respiratory syndrome coronavirus 2 (SARS-CoV-2) has reached over 533 million cases, with approximately 6.3 million deaths across the globe (1). Individuals who have recovered from the coronavirus disease (COVID-19) generate variable immune responses and remain at risk of reinfection (2). Several prior studies reported associations between age, comorbidities, and the severity of the acute infection with the magnitude and durability of the immune response (3, 4). This can be seen in several studies linking increasing disease severity with more robust humoral and cellular immune responses, suggesting that the immune response to natural infection is highly variable (5–7).

Reinfection with SARS-CoV-2 has been defined as a second positive RT-PCR test at least 90 days apart from the first infection, for which prolonged shedding of SARS-CoV-2 or viral RNA has been ruled out (8). Ideally, respiratory specimens from both episodes would be available to sequence to determine the different clades of SARS-CoV-2 (9). Patients who have recovered from COVID-19 have a lower risk of reinfection (8), though as the COVID-19 pandemic continues, reinfection has become increasingly common (10, 11). Since August 2020, few cases of COVID-19 reinfection have been reported with genomic data from paired episodes, thus making it challenging to establish true reinfection caused by different variants vs. persistent infection (2).

The scientific understanding of SARS-CoV-2 reinfection is still developing (12). Waning immunity from a previous infection and the emergence of new variants might negatively affect the protection provided by infection-acquired immunity, ultimately leading to the occurrence of reinfections (13). Related to waning immunity, Townsend et al. estimated that the time to reinfection by SARS-CoV-2 under endemic conditions would likely occur between 3 and 63 months after peak antibody response, with a median of 16 months (10). Since November 2021, five SARS-CoV-2 variants have been designated as Variants of Concern (VOC): Alpha (Pango Lineage B.1.1.7), Beta (Pango Lineage B.1.351), Gamma (Pango Lineage have since circulated P.1), Delta (Pango Lineage B.1.617.2), and Omicron (Pango Lineage B.1.1.529). These VOCs have since circulated worldwide (14). Among those variants, data indicate that Delta, which was first identified in India in December 2020 and rapidly spread globally, is associated with enhanced pathogenicity, can cause more severe disease, is 40–60% more transmissible than Alpha and is almost twice as transmissible as the original Wuhan strain of SARS-CoV-2 (15–17). The emergence of these variants has raised concerns about the breadth of neutralizing and cross-protective antibody responses induced by previous infection (18). To date, there have been few reports on sequential SARS-CoV-2 infections with supporting molecular evidence (2, 19), including sequence data. Here we report a confirmed reinfection case in Indonesia, with the first infection caused by Pango lineage B.1.466.2 and the second infection, 6 months later, caused by the Delta VOC.

## Case description

**FIGURE 1:**
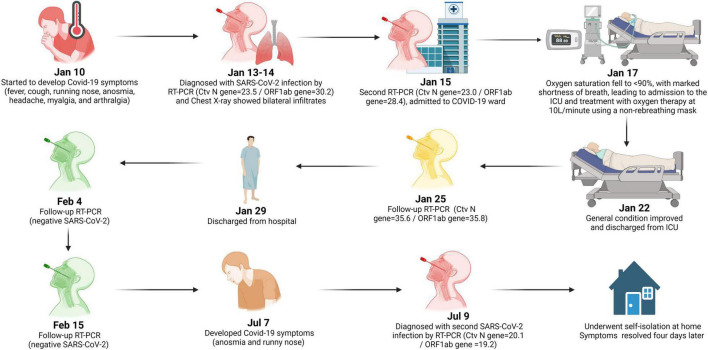
Timeline of reinfection case. Image was created with BioRender.com.

A summary reinfection case timeline is presented in Figure 1. A 49-year-old male resident of Tangerang, Banten, Indonesia, worked as a driver and utility worker at Tangerang District Hospital. He lived alone in a private room and commuted every day to the hospital on public transportation. He had not received COVID-19 vaccination, and his only known chronic condition was hypertension, for which he received no routine treatment. On January 10, 2021, the patient developed clinical symptoms consistent with COVID-19, including fever, cough, runny nose, anosmia, headache, myalgia, and arthralgia. On January 13, 2021, the patient was admitted to the emergency department. Physical examination revealed the patient was fully conscious, with high blood pressure (175/110 mmHg), fever (38°C), respiratory rate of 22 breaths/min, heart rate of 98 beats/min, and oxygen saturation of 97%. Nasopharyngeal (NP) and oropharyngeal (OP) swabs were collected for RT-PCR testing, and the following day he was diagnosed with SARS-CoV-2 infection (Ct values: Nucleocapsid (N) gene = 23.5 and ORF1ab gene = 30.2) but remained in the emergency department due to overloaded hospital COVID-19 wards. An initial chest X-ray showed bilateral pulmonary infiltrates, and his laboratory tests showed elevated C-reactive protein (CRP) (66.3 mg/L), elevated D-dimer (241 ng/mL), leukocytosis (10.9 × 10^3^/μL) with neutrophil-to-lymphocyte ratio (NLR) of 9.6, and mild thrombocytopenia (134 × 10^3^/μL). The patient was admitted to the COVID-19 ward on January 15 and received favipiravir, methylprednisolone, antihypertensives, and vitamin C. A repeated RT-PCR test showed that he was still positive (Ct values: N-gene = 23.0 and ORF1ab gene = 28.4). A summary of serial blood tests performed during hospitalization is shown in Table 1.

**TABLE 1:** Blood laboratory testing during infection episode 1.

No.	Parameter	Reference range	Units	Test date						
				Jan 14, 2021	Jan 15, 2021	Jan 17, 2021	Jan 20, 2021	Jan 21, 2021	Jan 25, 2021	Jan 28, 2021
**1**	**Hematology**									
	Hemoglobin	13.2–17.3	g/dL	16.9	–	15.1	16.1	–	16.6	–
	Leukocyte	3.8–10.6	×10^3^/μL	9.49	–	10.90	11.94	–	15.15	–
	Hematocrit	40–52	%	46	–	43	45	–	48	–
	Thrombocyte	140–440	×10^3^/μL	134	–	238	372	–	511	–
**2.**	**Differential count**									
	Basophil	0–1	%	0	–	–	0	–	0	–
	Eosinophil	2–4	%	0	–	–	0	–	0	–
	Band neutrophil	3–5	%	0	–	–	0	–	0	–
	Segmented neutrophil	50–70	%	86	–	–	86	–	78	–
	Lymphocyte	25–40	%	9	–	–	6	–	12	–
	Monocyte	2–8	%	5	–	–	8	–	10	–
**3.**	**Hemostasis**									
	Prothrombin time	12.8–15.9	Seconds	–	16.2	–	–	–	–	–
	INR	<1.10	–	–	1.13	–	–	–	–	–
	aPTT	21.0–53.0	Seconds	–	34.3	–	–	–	–	–
	d-dimer	<200	ng/mL	–	241	–	–	212	–	158
**4.**	**Blood chemistry**									
	C-Reactive protein	≤5	mg/L	–	66.34	–	14.16	–	–	–
	Interleukin 6 (IL-6)	≤7.0	pg/mL	–	–	–	–	2.92	–	–
	Blood glucose	<180	mg/dL	149	–	–	–	–	–	–
	Urea	0–50	mg/dL	–	–	–	–	–	–	–
	Creatinine	0.0–1.3	mg/dL	–	–	–	–	–	–	–
	NT-Pro BNP	<125	pg/mL	–	–	–	–	< 50	–	–
**5.**	**Electrolyte**									
	Natrium	135–147	mEq/L	–	–	–	–	131	–	–
	Potassium	3.5–5.0	mEq/L	–	–	–	–	4.9	–	–
	Chloride	96–105	mEq/L	–	–	–	–	92	–	–
**6.**	**Blood gas analysis**									
	pH	7.35–7.45	–	–	–	7.457	–	7.454	–	–
	PCO_2_	35.0–45.0	mmHg	–	–	30.3	–	41.0	–	–
	PO_2_	80–100.0	mmHg	–	–	120.5	–	63.9	–	–
	HCO_3_	22.0–26.0	mmHg	–	–	22.2	–	28.3	–	–
	Base excess (BE)	–2.5 to 2.5	mmol/L	–	–	–3.0	–	4.3	–	–
	O_2_ Saturation	96.0–97.0	%	–	–	98.7	–	93.8	–	–

INR, international normalized ratio; aPTT, activated partial thromboplastin time; NT-Pro BNP, N-terminal prohormone of brain natriuretic peptide; pH, potential of hydrogen; PCO_2_, partial pressure of carbon dioxide; PO_2_, partial pressure of oxygen; HCO_3_, bicarbonate.

On January 17, the patient’s oxygen saturation fell to 88% with marked shortness of breath, leading to his admission to the ICU and treatment with oxygen therapy at 10L/minute using a non-rebreathing mask. While in the ICU, his antiviral treatment was changed to a 5-day course of remdesivir and meropenem, per the attending physician’s clinical judgment. No bacterial culture and antimicrobial susceptibility testing (AST) were performed. He also received heparin, which was then switched to fondaparinux sodium due to increased risk of bleeding, and a furosemide injection due to high blood pressure (up to 163/97 mmHg). After 3 days in the ICU, his condition improved, and his oxygen therapy was weaned to a nasal cannula. On January 22, the patient was moved to the regular COVID-19 ward, received oral levofloxacin for 5 days, and continued with oral cefixime, per clinical judgment due to an elevated leukocyte count (15.2 × 10^3^/μL). A secondary bacterial infection was never confirmed. A follow-up RT-PCR test on January 25 detected low levels of viral RNA (Ct values: N-gene = 35.6 and ORF1ab gene = 35.8). The patient met discharge criteria [i.e., 10 days after onset of symptoms and at least three additional days without symptoms (20)] on January 29 and left the hospital. A summary of the patient’s treatments is shown in Table 2.

**TABLE 2:** Timeline of treatments provided during infection episode 1.

No.	Treatment (route)	Dosage	Duration	Timeline																					
				Pre-ICU				ICU					Post-ICU								Post-discharge				
				January																			February		
				13	14	15	16	17	18	19	20	21	22	23	24	25	26	27	28	29	30	31	1	2	3
1	Favipiravir (oral)	Day 1 = 2 × 1600 mg Day 2–5 = 2 × 600 mg	2 Days																						
2	Remdesivir (iv)	Day 1 = 1 × 200 mg Day 2–5 = 1 × 100 mg	5 Days																						
3	Oxygen (NRM)	10 Lpm	4 Days																						
4	Oxygen (nasal canule)	2–4 Lpm	8 Days																						
5	Metylprednisolone (iv)	2 × 32.5 mg	13 Days																						
6	Heparin (sc)	2 × 5,000 Unit	3 Days																						
7	Fondaparinu × sodium (sc)	1 × 2.5 mg	10 Days																						
8	Meropenem (iv)	3 × 1,000 mg	8 Days																						
9	Levofloxacin (oral)	1 × 750 mg	5 Days																						
10	Cefixime (oral)	2 × 200 mg	7 Days																						
11	Vitamin C (iv)	3 × 500 mg	14 Days																						
12	Candesartan (oral)	2 × 16 mg	Maintenance																						
13	Hydrochlorothiazide (oral)	1 × 12.5 mg	Maintenance																						
14	Amlodipine (oral)	1 × 5 mg	14 Days																						
15	Furosemide (iv)	1 × 20 mg	10 Days																						
16	Paracetamol (oral)	3 × 500 mg as needed	1 Day																						
17	Ambroxol (oral)	3 × 30 mg as needed	Maintenance																						

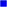
 Antivirus; 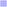 Oxygen therapy; 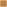 Steroid; 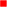 Anti-coagulant.

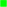
 Antibiotic; 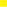 Vitamin; 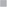 Antihypertensives; 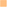 Symptomatic drugs.

iv, intravenous; sc, subcutaneous; NRM, non-rebreathing mask; Lpm, liter/minute.

**FIGURE 2:**
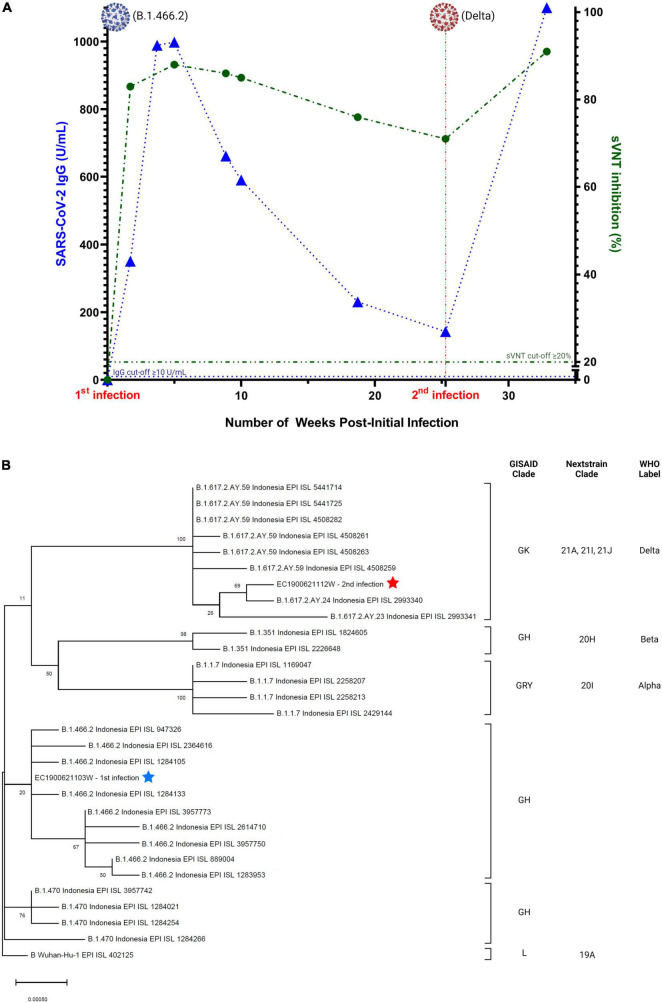
**(A)** Serial serology of SARS-CoV-2 IgG (blue) and neutralizing antibody (green). Image was created with GraphPad Prism 9.3.1 and BioRender.com, **(B)** phylogenetic analysis of SARS-CoV-2 genomes showing the relationship between the two distinct variants of two infection episodes. The tree was constructed by maximum likelihood method. Clade information as inferred by GISAID, Nextstrain, and Pangolin nomenclatures, are shown. The reference genome Wuhan-Hu-1 (GenBank accession number NC_045512.2) is used as the root of the tree.

On January 25, 2021, serum was collected and antibodies to the SARS-CoV-2 spike protein receptor binding domain (S-RBD) were detected by chemiluminescence immunoassay (CLIA) CL-900i^®^ SARS-CoV-2 S-RBD IgG (Mindray, Shenzhen, China), with an IgG index of 350.8 U/mL. Further testing using a surrogate virus neutralization test (sVNT) (GenScript, Piscataway, United States) resulted in a percent inhibition value of 83%. Following the patient’s discharge on January 29, follow-up RT-PCR tests on NP and OP specimens were performed on February 4 and 15, both of which were negative. Genomic sequencing of the SARS-CoV-2 Spike (S) gene from specimens collected on January 13, 2021, revealed S-D614G and S-N439K mutations. The full sequence of the S-gene (3,822-bp) was mapped and aligned to the reference genome obtained from the GISAID database (21). The sequence showed a 100% similarity with accession ID: EPI_ISL_1284133, belonging to Pango Lineage B.1.466.2 or GH clade on GISAID (22). Subsequent serological testing was performed at several timepoints after the initial COVID-19 episode (Figure 2A), with a peak S-RBD IgG index of 998.4 U/mL and a peak sVNT value of 88% on February 17. By May 24, the S-RBD IgG index declined to 230.5 U/mL and sVNT to a value of 76%.

The second episode of SARS-CoV-2 infection occurred around July 7, 2021, during the surge of the Delta variant in Indonesia (23). The patient developed mild symptoms consistent with COVID-19, including anosmia and runny nose. Two days later, he underwent RT-PCR testing at Tangerang District Hospital and was positive for SARS-CoV-2 (Ct values: N-gene = 20.1 and ORF1ab gene = 19.2). No routine blood test and chest imaging were performed. S-gene sequencing revealed mutations in the S gene (T19R, G142D, E156G, Del157/158, A222V, L452R, T478K, D614G, P681R, and D950N) that were consistent with the Delta variant, belonging to Pango Lineage B.1.617.2 or clade 21A on Nextstrain (22). Genomic analysis showed that the first episode viral genome belonged to a different clade/lineage from the second viral genome (Figure 2B). The patient underwent self-isolation at home and received treatment with vitamins, oseltamivir, paracetamol, and candesartan. His symptoms resolved 4 days later, with no follow-up on RT-PCR test. Serology testing from a blood specimen collected on July 9, 2021, revealed low antibody levels (S-RBD IgG = 142.7U/mL and sVNT = 71%). Subsequent testing on August 31, 2021, demonstrated boosting of humoral immunity (S-RBD IgG > 1,000 U/mL and sVNT = 91%). Details of the laboratory assays are summarized in Supplementary Table 1.

## Discussion

Our case report documents the first confirmed SARS-CoV-2 reinfection case in Indonesia, though it is highly likely that there have been many instances of reinfection nationwide. We observed a patient with severe COVID-19 disease caused by the B.1.466.2 variant in the first episode followed by milder disease caused by the Delta variant approximately 6 months later. Serial serology testing showed a persistent but gradual decrease of SARS-CoV-2 antibody titers after the first episode, with a boost of humoral immunity following the second infection. Although we did not perform testing on fecal specimens between episodes to rule out prolonged viral shedding in the GI tract (24), this presented case met all criteria for a confirmed diagnosis of reinfection: (1) Confirmation of a true first episode (Ct values < 35), (2) proof of a reinfection with SARS-CoV-2 RT-PCR test with Ct < 35, (3) confirmation of infection with two different/distinct phylogenetic strains/variants, and (4) negative RT-PCR tests between the first and second episodes (8). Furthermore, the epidemiological and serological analysis also confirmed that our case was a true reinfection, in which the second episode occurred during the Delta wave in Indonesia, boosting the antibody levels from the prior infection without a history of vaccine administration.

The non-sterilizing immunity generated against SARS-CoV-2 infection has made it challenging to differentiate prolonged shedding from a true reinfection (25). The US CDC maintains that the best evidence of reinfection is differing clades/lineages/variants of SARS-CoV-2, as defined in Nextstrain and GISAID, between an initial and subsequent infection (9). Based on WHO guidelines, complete or partial S-gene sequences can be used to confirm infection with a specific variant (26). In this case report, we relied on targeted S-gene sequencing to determine viral variants. The sequencing results of the first virus showed 100% similarity with the B.1.466.2 virus lineage, which was reported to be an indigenous dominant strain in Indonesia before the Delta variant surge, with an exponential growth from October 2020 to February 2021 (27, 28). While our S-gene sequence fully aligns with B.1.466.2, we cannot be certain that the whole genome contains the additional expected mutations (with mutation frequencies > 99%) of ORF1a-T1168I, ORF1a-P1640L, ORF1b-P314L, ORF3a-Q57H, and N-T205I in the B.1.466.2 variant (28). The virus from the second episode can be definitively identified as the Delta variant due to the presence of specific Delta mutations in the sequenced S-gene (L452R, T478K, and P681R) (29).

Recovery from many viral diseases is followed by a period of infection-induced immunologic protection against reinfection (30). Antibodies against SARS-CoV-2 include both binding and neutralizing antibodies (31), with the antigenic targets most frequently assessed being the S and N proteins (30). IgM, IgA, and IgG isotypes may be produced against any of these antigens and can be detected in the blood starting from 5 to 15 days following symptom onset (32, 33). Both binding and neutralizing antibody titers rise faster and reach a higher peak in persons with more severe COVID-19 (4, 30). Several studies have suggested that the waning of antibody levels occurs within several months after infection (34, 35), and the incidence of subsequent SARS-CoV-2 reinfection may be inversely associated with baseline antibody titers (36). As shown in our report, IgG and neutralizing antibodies steadily declined after the first infection episode, were at their lowest levels just prior to the second infection and increased following reinfection. There are insufficient data to determine antibody titer thresholds indicative of an individual’s protection from infection, and the level of protection may not be the same for all viral variants (30). One analysis suggested that the antibody level associated with protection against severe disease is lower than the level associated with protection against infection (37).

Despite growing evidence that immune responses remain robust and protective against reinfection with antigenically similar variants by 80–90% for at least 5–7 months after primary infection (36, 38–40), our report supports the idea that the duration of protection may be shorter with new viral variants. In laboratory studies, decreased neutralization to Delta and other variants has been demonstrated in non-Delta convalescent and post-vaccination sera (41–43). Combined *in vitro* experimentation and epidemiology studies have highlighted that mutations in the Spike-RBD of the Delta variant may result in increased infectivity and reduced neutralization sensitivity to sera from individuals infected with prior variants (29, 44, 45). These escape mutations are thought to be implicated in reinfection (46), but the observed reduction in effectiveness has been modest, with continued strong protection against hospitalization, severe disease, and death (30). Persistent immunity from SARS-CoV-2 specific T cells may have an important role in reducing viral replication and limiting the pathogenicity of infection (47), though we did not examine T cell responses as part of this case.

Reports on the clinical severity of reinfection compared to primary infection vary (2, 19, 48). In most cases, reinfections have appeared to be mild, with lower rates of pneumonia, heart failure, and acute kidney injury compared to primary infection episodes (48). Our clinical observations and lab results suggest that natural immunity from the patient’s initial infection reduced the severity of disease during the subsequent infection with the Delta variant. It is important to point out that this is not universally the case in the setting of reinfection (49–51). Future studies of cases of reinfection are needed to provide a clear picture of how robust the quality, quantity, and durability of protective immunity elicited by natural infection with SARS-CoV-2 is and its role in altering the course of subsequent infection. Given the rapid evolution of the virus and highly variable levels of host protection, it may be difficult to draw strong generalizations.

The patient did not receive any COVID-19 vaccines before or between his first and second episodes of infection. During that time, due to limited supplies, Indonesian national policy for receiving the COVID-19 vaccine only included individuals with no comorbidities, blood pressure less than 140/90 mmHg and no prior infection with COVID-19 (52). Thus, our patient was not eligible to receive the vaccine following his initial infection. However, the policy has since changed; COVID-19 survivors with blood pressure under 180/110 mmHg who are at least 1-month post-infection are now eligible (53, 54). Currently, the patient has received two doses of the Pfizer-BioNTech vaccine (on September 10, 2021, and October 1, 2021) and a booster dose with the Moderna vaccine (on December 31, 2021).

Several studies have shown that strong immune response, termed “hybrid immunity,” arises from a combination of natural and vaccine-generated immunity (55). Vaccination of convalescent individuals boosted the humoral immune response to well above the threshold of neutralization for multiple variants (29). Furthermore, memory B cell and CD4 + T cell components directed toward multiple spike and non-spike regions appear to be enhanced during hybrid immunity (29, 55). A recent report from Israel found that people who had been infected with SARS-CoV-2 and then received one dose of the Pfizer-BioNTech messenger RNA (mRNA) vaccine were less likely to be reinfected than previously infected individuals who were unvaccinated [hazard ratio, 0.18 (95% CI, 0.15–0.20)] (56). The substantial growing body of evidence strongly indicates that vaccination after infection significantly enhances protection and further reduces the risk of reinfection against an array of circulating viral variants, including Delta (30, 56–58).

A major strength of this report was our serial documentation of RT-PCR and serologic testing during and between both episodes of infection. This is rarely documented in routine practice. However, our study has several limitations. First, this is a case study of one individual, and therefore no broad conclusions or recommendations can be drawn. Second, we only relied on targeted S-gene sequencing to determine viral variants, although it is still acceptable within WHO guidelines (26). Whole genome sequences would be valuable in determining exact variant/Pango lineage. Third, T-cell responses were not measured in this study. Future studies in confirmed reinfection cases may provide a better picture of how the different components of the adaptive immune response can prevent or limit reinfection (39, 59). Fourth, we did not perform the gold standard virus neutralization test (VNT) or plaque reduction neutralization test (PRNT), which requires biosafety level 3 (BSL-3) facilities. However, the chemiluminescent immunoassays and surrogate neutralization assay (sVNT) used in this study have demonstrated good concordance with the gold standard PRNT for SARS-CoV-2 antibody detection in human sera (60, 61). They thus can serve as valid alternative to the VNT test in neutralizing activity measurements.

## Conclusion

We reported a case of SARS-CoV-2 reinfection with distinct variants in Indonesia (8). The milder presentation in the second infection deserves further investigation to provide a clear picture of the role of post-infection immunity in altering the course of subsequent disease. As SARS-CoV-2 continues to circulate and mutate, vaccination following natural infection may prove to be an important strategy to increase protection against reinfection and disease.

## Ethics statement

Ethical review and approval was not required for the study on human participants in accordance with the local legislation and institutional requirements. The patients/participants provided their written informed consent to participate in this study. Written informed consent was obtained from the individual(s) for the publication of any potentially identifiable images or data included in this article.

## Author contributions

NI, FB, and DL performed clinical assessments and were responsible for data collection. DB-b, GS, and WW performed laboratory assays. YM, HK, DL, JP, AN, and HL reviewed data and wrote the manuscript. All authors contributed to the manuscript development, provided critical review, and have approved the final version.

## Conflict of Interest

JP was employed by Leidos Biomedical Research, Inc. The remaining authors declare that the research was conducted in the absence of any commercial or financial relationships that could be construed as a potential conflict of interest.

## Publisher’s Note

All claims expressed in this article are solely those of the authors and do not necessarily represent those of their affiliated organizations, or those of the publisher, the editors and the reviewers. Any product that may be evaluated in this article, or claim that may be made by its manufacturer, is not guaranteed or endorsed by the publisher.
