# Supplementary material for: Mild reinfection with severe acute respiratory syndrome coronavirus 2 (SARS-CoV-2) Delta variant: First case report from Indonesia
Source: Front Med (Lausanne). 2022 Jul 22;9:906469. doi: 10.3389/fmed.2022.906469 (PMC9355687; doi:10.3389/fmed.2022.906469)
Supplement: Supplementary file 1 [file Table_1.DOCX]

**Supplementary Table 1**. Summary of method details.

| **Assays** | **Procedures** |
| --- | --- |
| RT-PCR | For realtime RT-PCR-based detection, SARS-CoV2 RNA is extracted from 200 µl of NP using the Nextractor® NX-48S automated system (Genolution) (1). RT-PCR was performed using the Applied Biosystems 7500 Fast Realtime PCR Detection System (Thermo Fisher Scientific) and the 2019-nCoV Nucleic Acid Diagnostic Kit (Sansure Biotech, Inc.) detecting the N and ORF1ab genes of SARS CoV2 (2). The validity of assay in the kit is confirmed from detection of an internal control target, RNAse P gene. The reaction composition and condition follow the manufacturer’s recommendation. The PCR composition was a 25-µl mixture consisting of 13-µl of PCR mix, 2-µl of enzyme mix, and 10-µl of viral RNA, whereas the cycle condition was 50^o^ C reverse transcription for 30 minutes followed by an initial denaturation at 95^o^ C for 1 minute and 45 cycles of 95^o^ C for 15 seconds and 60^o^ C for 10 seconds. The Ct cut-off is ≤40. |
| Viral Genomic Sequencing | SARS-CoV2 RNA was extracted from 140 µl of RT-PCR positive NP specimen using the QIAamp Viral RNA Mini Kit (Qiagen). The sequencing protocol follows a method targeting the whole S gene (SARS-CoV2 genomic position 21563 – 25384, 3822-bp) developed by Dr. W. Ian Lipkin’s Laboratory (Columbia University – please check the affiliation) (3). Firstly, the S-gene segment was amplified in 8 overlapping fragments using One-Step RT-PCR Kit (Qiagen) and its suggested protocol; each was a 25-µl reaction consisting of 5-µl 5X One-Step RT-PCR Mix, 1-µl of 10 mM dNTP, 1-µl of each primer (10 µM), 11-µl of nuclease-free water, and 5-µl of RNA, with the following cycle condition: reverse transcription at 50^o^ C for 30 minutes, initial denaturation of 95^o^ C for 15 minutes, 35 cycles of 94^o^ C for 30 seconds, 55^o^ C for 30 seconds, and 72^o^ C for 1 minute, and a final extension at 72^o^ C for 10 minutes. The RT-PCR amplicons were visualized in 1.5% agarose gel electrophoresis, where F1 = 681-bp, F2 = 551-bp, F3 = 550-bp, F4 = 671-bp, F5 = 572-bp, F6 = 715-bp, F7 = 658-bp, F8 = 541-bp. Prior to entering cycle sequencing, the RT-PCR amplicons were purified with ExoSAP-IT™ Express PCR Product Cleanup Reagent (Thermo Fisher Scientific), with the reaction composition consisting of 5-µl RT-PCR product and 2-µl ExoSAP whereas the reaction condition was 4-minutes clean-up at 37^o^ C and 1-minute inactivation at 80^o^ C.  We performed cycle sequencing using the BigDye Terminator v3.1 Cycle Sequencing Kit (Thermo Fisher Scientific) as per manufacturer’s protocol (4), where the reaction composition was 2-µl of 2.5X BigDye Terminator v3.1 Ready Reaction Mix, 1-µl of 5X BigDye Terminator v1.1/3.1 Sequencing Buffer, 0.5-µl of 3.2 µM primer, 2-µl of purified RT-PCR product, and 4.5-µl of nuclease-free water. Cycle sequencing condition was performed in a Proflex (Thermo Fisher Scientific) thermal cycler, consisting of an initial denaturation at 96^o^ C for 1 minute and 25 cycles of 96^o^ C for 10 seconds, 50^o^ C for 30 seconds, and 60^o^ C for 4 minutes. We purified 10- µl of cycle sequencing product using BigDye XTerminator^TM^ Purification Kit (Thermo Fisher Scientific), with a composition of 10-µl of XTerminator^TM^ Solution, 45-µl of SAM^TM^ Solution, and a mixing condition of 2000 rpm, 4^o^ C for 40 minutes. The base composition in purified cycle sequencing product was electrophoresed in a capillary electrophoresis system Genetic Analyzer (GA) 3500 (Thermo Fisher Scientific) using FastSeq50_POP7 assay in the 3500 Series Data Collection Software 3.3. The outcome is an .ab1 file that nucleotides were basecalled using Sequencing Analysis Software 7 (Thermo Fisher Scientific). Chromatograms of the sequencing reads (F1 – F8) were edited and joined to retrieve the S-gene DNA sequence (Sequencher 5.4.6 (Genecodes)).  The identity of the S gene was determined by submitting the sequence to GISAID database, to retrieve the highest similarity of nucleotides, mutation list, lineage (Pango), clade (Nextstrain), and variant (WHO). |
| Phylogenetic Analyses | We analyzed the Spike gene of SARS-CoV-2 (21,563 - 25,384; numbered according to the reference genome Wuhan-Hu-1 (GenBank accession number MN908947.3 or GISAID identifier IEPI_ISL_402125). Multiple alignments were performed using ClustalW (5). A maximum-likelihood phylogenetic tree was constructed using MEGA V7.0.26 (6), with substitution model T92+G as the best-predicted model by BIC and 100 bootstrap replicates. We described the clade information using GISAID (7) and Pangolin (8) nomenclatures. The reference sequences were retrieved from the GISAID database from strains that circulated from January 1^st^ to August 31^st^, 2021, in Indonesia (GH.B.1.466.2, GH.B.1.470, GRY.B.1.1.7, GH.B.1.351, GK.B.1.617.2.AY.59, GK.B.1.617.2.AY.23, and GK.B.1.617.2.AY.24). Sequences having N nucleotide in the S gene were excluded from the analysis. The tree was rooted with Wuhan-Hu-1 reference genome. |
| Serology Assay – SARS-CoV-2 IgG S-RBD chemiluminescence immunoassay (CLIA) | The commercial CLIA on automated analyzers, CL-900i® SARS-CoV-2 IgG S-RBD (Cat. No. SARS-CoV2 IgG121, Mindray, Shenzhen, China), was used for the semi-quantitative determination of specific IgG antibodies to receptor-binding domain (RBD) of SARS-CoV-2 spike protein in patient’s serum (9). The test was carried out according to the manufacturer’s instructions. In brief, quantitative detection of IgG is completed in a two-step assay. In the first step, sample, sample treatment solution, paramagnetic microparticles coated with S-RBD SARS-CoV-2 antigens are added to a reaction vessel. After incubation, S-RBD SARS-CoV-2 IgG antibodies in the sample will bind to S-RBD SARS-CoV-2 antigen-coated microparticles. Afterward, microparticles are magnetically captured while other unbound substances are removed by washing. In the second step, diluent solution, ALP labeled anti-human IgG monoclonal antibodies are added to the reaction vessel. After incubation, ALP labeled anti-human IgG monoclonal antibody will form a sandwich structure with microparticle captured S-RBD SARS-CoV-2 IgG antibodies. Afterward, microparticles are magnetically captured while other unbound substances are removed by washing. Then, the substrate solution is added to the reaction vessel. It is catalyzed by anti-human IgG antibody-ALP conjugate in the immune-complex retained on the microparticles. The resulting chemiluminescent reaction is measured as relative light units (RLUs) by a photomultiplier built into the system. The amount of S-RBD SARS-CoV-2 IgG antibodies present in the sample is proportional to the relative light units (RLUs) generated during the reaction. Specimens with results of ≥10.00 U/mL are considered positive for IgG antibodies to S-RBD-SARS-COV-2, suggesting previous or recent infection |
| Serology Assay – surrogate virus neutralization test (sVNT) | The SARS-CoV-2 sVNT Kit (L00847; GenScript, Piscataway, USA) utilizes the recombinant RBD of the SARS-CoV-2 spike protein to detect antibodies that block the RBD from binding to the hACE2 receptor (10). The assay was performed according to manufacturer instructions, with serum samples and the kit-supplied positive and negative controls were diluted 1:10 in kit-specific sample dilution buffer. The diluted samples and controls were preincubated with horseradish peroxidase-conjugated RBD (HRP-RBD) at 37°C for 30 minutes. The mixtures were added to the hACE2-coated capture plate and incubated at 37°C for 15 minutes. Plates were then washed, removing HRP-RBD: neutralizing antibody complexes and allowing unbound HRP-RBD and HRP-RBD:non-neutralizing antibody complexes to remain bound to hACE2. After the wash steps, 3,3’,5,5’-tetramethylbenzidine (TMB) solution was added and allowed to incubate at room temperature for 15 minutes and the reaction was stopped by Stop Solution. The OD of each well was measured by spectrophotometry at 450 nm. The color intensity is inversely proportional to the amount of neutralizing antibody in standards or samples. The percent inhibition of a sample was calculated as (1-Average OD of sample/Average OD of negative control) x 100%. A sample with a percent inhibition of <20% was considered “negative”, and ≥20% was considered “positive” for SARS-CoV-2 neutralizing antibodies, which blocking the RBD-hACE2 interaction. |

***Footnote References:***

1. Genolution. Nextractor® NX48S User Guide [Internet]. 2020 [cited 2022 Jun 17]. p. 1–23. Available from: https://dmec.moh.gov.vn/documents/10182/22203828/upload_00009943_1631869394843.pdf?version=1.0&fileId=22226000

2. Biotech S. Novel Coronavirus (2019-nCoV) Nucleic Acid Diagnostic Kit (PCR-Fluorescence Probing) [Internet]. US Food and Drug Administration website. 2020 [cited 2022 Jun 17]. p. 1–27. Available from: https://www.fda.gov/media/137651/download

3. Andersen KG, Rambaut A, Lipkin WI, Holmes EC, Garry RF. The proximal origin of SARS-CoV-2. Nat Med [Internet]. 2020;26(4):450–2. Available from: https://doi.org/10.1038/s41591-020-0820-9

4. Biosystems A. BigDye Terminatior v3.1 Cycle Sequencing Kit [Internet]. Vol. 20, Plant Molecular Biology Reporter. 2002 [cited 2022 Jun 17]. p. 187–187. Available from: https://assets.thermofisher.com/TFS-Assets/LSG/manuals/cms_081527.pdf

5. Thompson JD, Higgins DG, Gibson TJ. CLUSTAL W: improving the sensitivity of progressive multiple sequence alignment through sequence weighting, position-specific gap penalties and weight matrix choice. Nucleic Acids Res. 1994 Nov;22(22):4673–80.

6. Kumar S, Stecher G, Tamura K. MEGA7: Molecular Evolutionary Genetics Analysis Version 7.0 for Bigger Datasets. Mol Biol Evol. 2016 Jul;33(7):1870–4.

7. Khare S, Gurry C, Freitas L, Schultz MB, Bach G, Diallo A, et al. GISAID’s Role in Pandemic Response. China CDC Wkly [Internet]. 2021;3(49):1049–51. Available from: https://doi.org/10.46234/ccdcw2021.255

8. Rambaut A, Holmes EC, O’Toole Á, Hill V, McCrone JT, Ruis C, et al. A dynamic nomenclature proposal for SARS-CoV-2 lineages to assist genomic epidemiology. Nat Microbiol. 2020 Nov;5(11):1403–7.

9. Cristiano A, Pieri M, Sarubbi S, Pelagalli M, Calugi G, Tomassetti F, et al. Evaluation of serological anti-SARS-CoV-2 chemiluminescent immunoassays correlated to live virus neutralization test, for the detection of anti-RBD antibodies as a relevant alternative in COVID-19 large-scale neutralizing activity monitoring. Clin Immunol. 2022 Jan;234:108918.

10. M. PRAP, Ronald K, Y. TOT, C. HDS, M. KMY, J. BC, et al. Evaluation of a SARS-CoV-2 Surrogate Virus Neutralization Test for Detection of Antibody in Human, Canine, Cat, and Hamster Sera. J Clin Microbiol [Internet]. 2022 Jun 14;59(2):e02504-20. Available from: https://doi.org/10.1128/JCM.02504-20
